# Steatotic liver disease associated with 2,4-dienoyl-CoA reductase 1 deficiency

**DOI:** 10.1038/s41366-024-01634-z

**Published:** 2024-09-14

**Authors:** Benno Kohlmaier, Kristijan Skok, Carolin Lackner, Greta Haselrieder, Thomas Müller, Sabrina Sailer, Johannes Zschocke, Markus A. Keller, A. S. Knisely, Andreas R. Janecke

**Affiliations:** 1 Department of General Paediatrics, Medical University of Graz, 8010 Graz, Austria; 2 Diagnostic and Research Institute of Pathology, Medical University of Graz, 8010 Graz, Austria; 3 Department of Paediatrics I, Medical University of Innsbruck, 6020 Innsbruck, Austria; 4 Institute of Human Genetics, Medical University of Innsbruck, 6020 Innsbruck, Austria

**Keywords:** Translational research, Metabolic syndrome

## Abstract

**Background:**

Metabolic dysfunction-associated steatotic liver disease (MASLD) is considered multifactorial with a number of predisposing gene polymorphisms known.

**Methods:**

The occurrence of MASLD in 7 and 10 year old siblings, one without classical risk factors and one with type 2 diabetes suggested a monogenic etiology and prompted next-generation sequencing. Exome sequencing was performed in the proband, both parents and both siblings. The impact of a likely disease-causing DNA variant was assessed on the transcript and protein level.

**Results:**

Two siblings have hepatomegaly, elevated serum transaminase activity, and steatosis and harbor a homozygous *DECR1* splice-site variant, c.330+3A>T. The variant caused *DECR1* transcript decay. Immunostaining demonstrated lack of DECR1 in patient liver.

**Conclusions:**

These patients may represent the first individuals with DECR1 deficiency, then defining within MASLD an autosomal-recessive entity, well corresponding to the reported steatotic liver disease in *Decr1* knockout mice. *DECR1* may need to be considered in the genetic work-up of MASLD.

## Introduction

Hepatic lipid storage with chronic liver disease, or steatotic liver disease (SLD), common in children in the developed world, is observed in the setting of obesity, insulin resistance, and a sedentary lifestyle. Also called metabolic dysfunction-associated SLD (MASLD) [1], it may occur with or without recognized cardiometabolic risk factors [2]; in the latter setting, the terms “cryptogenic SLD” or “possible MASLD” are used [3]. Risk alleles for MASLD are known [4, 5]; the GG genotype at the rs738409 site of the patatin-like phospholipase domain containing gene 3 (*PNPLA3*) is both the most common and the most potent variant, doubling risk for developing MASLD [6–8]. MASLD comprises a spectrum of severities ranging from benign mostly non-progressive metabolic syndrome-associated steatotic liver to metabolic syndrome-associated steatohepatitis (MASH) [3].

Hepatic lipid storage is also common and highly prevalent in inherited monogenic enzyme deficiencies that constitute the fatty acid oxidation disorders [9–11]. To the best of our knowledge, we report the first instance of mitochondrial 2,4-dienoyl-CoA reductase (DECR1) deficiency, associated with a MASLD-spectrum phenotype in two siblings. DECR1 processes enoyl-CoA for re-entry into the β-oxidation cycle [12–14]. Reported *Decr1* knockout mice manifested fasting-related hypoglycemia, hepatic microvesicular steatosis, an altered fatty acid profile in liver and serum, and inability to maintain a normal body temperature during cold exposure [15, 16].

## Clinical and laboratory findings

We report 3 sibling children with age-appropriate neurodevelopment, born in Afghanistan to parents who are second cousins.

**Fig. 1:**
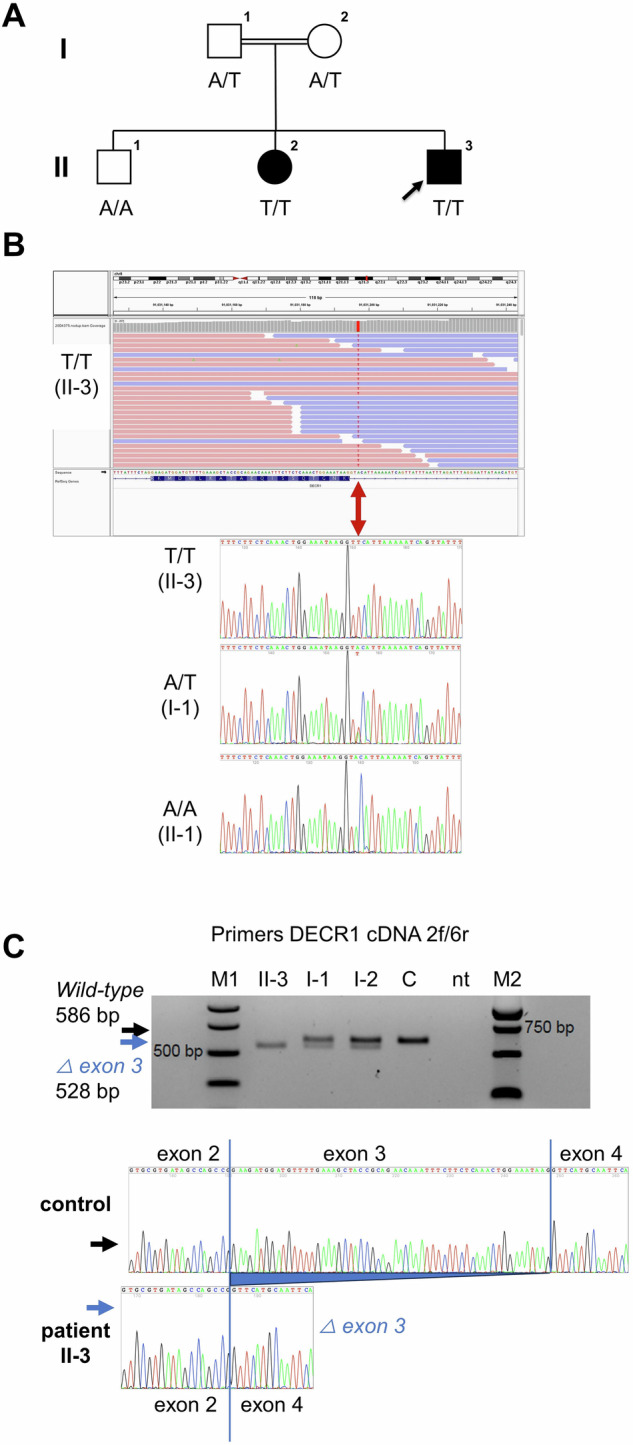
*DECR1* variant identification and transcript analysis. **A** The patients’ parents (I-1, I-2) are cousins and heterozygous for the *DECR1* variant c.330+3A>T **B** Their children II-2, II-3 have MASLD and are homozygous for this variant; the third (II-1) is homozygous for the wild-type (WT) allele. He has steatosis of the liver without fibrosis and without abnormality of serum biomarkers used to indicate hepatobiliary disease. **C** *DECR1* c.330+3A>T variant leads to an aberrant transcript (left panel) that lacks intron 3 (right panel), which undergoes nonsense-mediated decay.

Pregnancy and birth of the propositus (Fig. 1A, Patient II-3) were uneventful. Aged 6 y, he was referred for investigation of persistently elevated serum transaminase concentrations. Right upper eyelid xanthelasma was noted. His liver was enlarged (14 cm, right midclavicular line; mean 10.9 cm / SD 1.17 cm) and round-edged, with parenchymal inhomogeneity. Aged 10 y, he is of normal height and weight (Table S1), and continues to exhibit hepatomegaly and elevated transaminase values. Synthetic function is not impaired and there are is no indication of portal hypertension. On 24 h fasting he exhibited no signs of hypoglycemia (Table S2) and did not complain of distress. Insulin levels were not determined.

II-2, the sister of the propositus, at age 7 y was diagnosed with cervical Burkitt lymphoma, stage II, and complete remission was achieved within 5mo of treatment. She was overweight, with normal abdominal sonography and clinical-biochemistry test results. Hepatobiliary-injury biomarker values were elevated throughout her chemotherapy and normalized at the end of treatment, but rose again only 3mo thereafter; they continue to be high. Glycosylated hemoglobin concentrations were elevated and diabetes mellitus type II was diagnosed subsequent to her chemotherapy. At 13 y, she is a person with class I obesity (Supplemental Table S1). She takes metformin and liraglutide. Abdominal sonography shows hepatomegaly with hyperechogenicity; the liver edges are rounded and fat deposits were present in the hepatic capsule.

The brother of patients II-3 and II-2, II-1, aged 16 y, is a person with class II obesity. Abdominal sonography has found elevated liver brightness, interpreted as steatosis, with normal liver size (14 cm, right midclavicular line; mean 13.4 cm / SD 1.95 cm). Serum transaminases were not elevated and synthetic function was intact (Supplemental Table S1).

A diagnosis of DECR1 deficiency was made in the two younger siblings, whose phenotypes all fit into the MASLD spectrum.

## Materials and methods

Processing, staining, and immunohistochemical studies of liver-biopsy specimens from propositus and control are described in the Supplement. Genomic DNA was extracted from peripheral blood samples using standard procedures. Exome sequencing was performed with genomic DNA samples from the propositus, both parents, and both of his siblings. Whole exome capture, sequence data analysis, and variant validation by Sanger sequencing and classification are described in the Supplement. *DECR1* transcript analysis in the propositus, his parents, and a control is described in the Supplement. Plasma acylcarnitines were measured as described in the Supplement.

## Results

### Histopathologic findings, core liver biopsy of the propositus

**Fig. 2:**
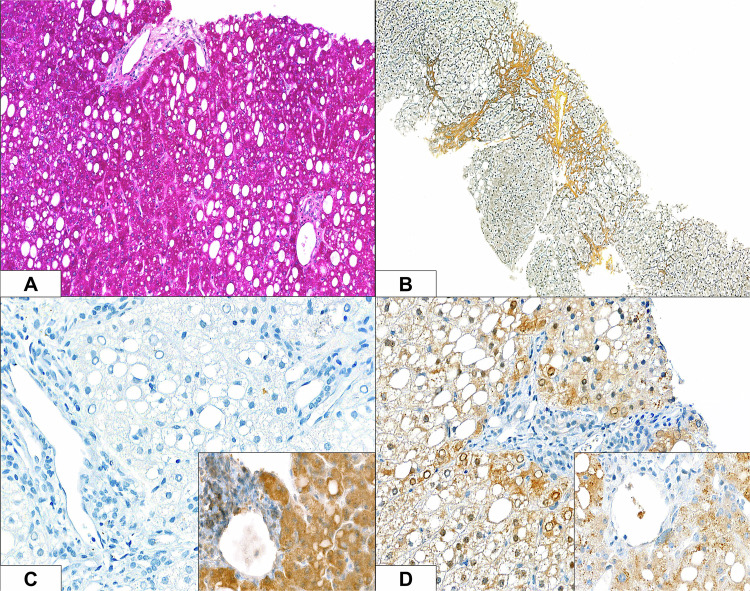
Liver, Patient 1, with immunohistochemical controls (insets). Macrovesicular steatosis **A** is manifest as vacuolation on staining with periodic acid–Schiff technique. Gömöri reticulin staining demonstrates mild portal-tract fibrosis and focal portal–portal bridging fibrosis **B**. Immunostaining for the antigens DECR1 and its homologue DECR2, with diaminobenzidine chromogen and haematoxylin counterstaining, identifies uniform lack of expression of DECR1 **C** and granular cytoplasmic expression of DECR2 **D**; same-slide control liver sections exhibit a granular marking pattern for both antigens. (Original magnifications 200x **A**, 100x **B**, and 400x **C** and **D**).

Macrovesicular steatosis (Fig. 2A) and chronic portal and lobular hepatitis with minimal activity were seen, with mild portal-tract fibrosis and focal portal–portal bridging fibrosis (Fig. 2B). Copper stores were not found. Mitochondria were unremarkable on ultrastructural study. Immunostaining for the antigens DECR1 and its homolog DECR2 demonstrated uniform lack of expression of DECR1 (Fig. 2C) and granular cytoplasmic expression of DECR2 (Fig. 2D) in the propositus’ liver; same-slide control liver sections displayed a granular pattern for both antigens.

### Plasma acylcarnitine profile

During a 24-h fast of the propositus, we observed the anticipated time-dependent increase in plasma 3-hydroxybutyrylcarnitine, along with a general release of longer-chain beta-oxidation intermediates. No segregation between saturated and polyunsaturated species was found.

### Identification of a DECR1 loss-of-function mutation in the family

Exome sequencing in the propositus revealed homozygosity for a variant within the canonical exon 3-intron 3 splice site of the *DECR1* gene, denoted NG_008042.2(NM_001359.2):c.330+3A>T. This variant is rare (gnomAD population database v4.0.0 allele frequency: 16 in 1,592,248 (0.001%), in heterozygous state only). Homozygosity for this variant was also found in the sister (II-3), whereas the brother (II-1) with obesity and hepatic steatosis, but without hepatomegaly and with normal biomarker values, is homozygous for the wild-type allele (Fig. 1A, B). Both parents are heterozygotes.

The propositus lacked wild-type transcript; minor amounts of an aberrant transcript lacking exon 3 of *DECR1* were seen in peripheral-blood leukocytes. The aberrant transcript encodes a frameshift and a premature stop codon (p.Lys92Phefs*8), subject to nonsense-mediated mRNA decay. Wild-type and aberrant *DECR1* transcripts were present in heterozygotes (Fig. 1C).

Known human fatty acid oxidation disorders or hepatopathies were identified in exome data in neither the propositus nor his siblings. Variants associated with MASLD in genome-wide analyses [4] were identified in the exome data and Supplemental Table S3 presents their segregation within this family, together with clinical risk factors.

## Discussion

Discovery of marked mixed steatosis of hepatocytes, with fibrosis and inflammation, on histopathologic study of liver of a 7 y old boy with lean BMI and elevated transaminases prompted extended genetic testing. This identified a homozygous loss-of-function variant in *DECR1*. Immunohistochemical study of liver-biopsy materials confirmed absence of DECR1 protein. The same variant in homozygous form was found in the propositus’ sister with elevated BMI, elevated transaminase values, and sonographic evidence of steatotic hepatomegaly indicative of MASLD; her evaluation was complicated by completed non-Hodgkin lymphoma treatment. Neither hepatomegaly nor abnormal transaminase values were present in their elder brother with class II obesity and sonographic evidence of hepatic steatosis who does not carry the *DECR1* variant. The clinical and genetic observations in this family suggest that autosomal-recessive deficiency of DECR1 may represent a novel cause of MASLD. This conclusion is supported by the clinical findings in *Decr1* knockout mice, which developed microvesicular SLD upon fasting, hypoglycemia under metabolic stress [15], and fatal hypothermia upon acute cold challenge [16].

Genetic DECR1 deficiency has not been described in humans; secondary DECR deficiency was observed twice in nicotinamide adenine dinucleotide kinase deficiency [17, 18]. In *Decr1*-deficient mice, levels of acylcarnitine moieties in serum were increased, especially decadienoylcarnitine, a product of the incomplete oxidation of linoleic acid (C18:2). Urinary excretion of unsaturated dicarboxylic acids also occurred after 24 h fasting. None of these abnormalities was detectable in the *DECR1*-deficient propositus in the present family, who maintained normal serum glucose level, ketogenesis, and acid-base balance during fasting for 24 h. An extended acetylcarnitine profile did not show abnormal metabolites; the carnitine C10:2 ratio was normal. Our data suggest that in humans, DECR1 plays only a minor role in the catabolism of fatty acids released by lipolysis during fasting. There is no evidence that the proportion of polyunsaturated fatty acids during fasting is sufficiently high to give rise to the accumulation of specific pathological metabolites in DECR1 deficiency, which would confer a risk for acute metabolic events during fasting. However, accumulation of long polyunsaturated fatty acids of decreased β-oxidation may hypothetically underlie SLD here.

The relevance and frequency of DECR1 deficiency in the development of MASLD remain to be clarified. DECR1 deficiency may potentially lead to MASLD only in combination with additional SLD risk factors such as the *PNPLA3* Ile148Met variant. According to the gnomAD population variant database, loss-of-function variants in *DECR1* are observed at expected rates given the length and sequence composition of this gene, without evidence of selection against heterozygotes. Considering that the MASLD spectrum in our patients is relatively mild, other similarly affected individuals may have failed to qualify to complete genetic work-up. This could possibly explain why DECR1 deficiency has not been proposed as a cause of MASLD. MASLD can be associated with a variety of cardiometabolic, orthopedic and psychological complications already in childhood and adolescence [19]; considering the obesity pandemic with a worldwide obesity rate of approximately 40% in adults [20], and increased overall genetic testing, DECR1 deficiency may be more likely to be recognized in the future.

## Supplementary information

Supplemental Methods and Results

## Data Availability

All relevant data generated or analysed during this study are included in this published article and its supplementary information files.
