# Supplementary material for: Steatotic liver disease associated with 2,4-dienoyl-CoA reductase 1 deficiency
Source: Int J Obes (Lond). 2024 Sep 14;48(12):1818–21. doi: 10.1038/s41366-024-01634-z (PMC11584395; doi:10.1038/s41366-024-01634-z)
Supplement: Supplementary file 1 — Supplemental Methods and Results [file 41366_2024_1634_MOESM1_ESM.docx]

*SUPPLEMENT TO*

**Steatotic liver disease associated with 2,4-dienoyl-CoA reductase 1 deficiency**

Benno Kohlmaier^1^, Kristijan Skok^2^, Karoline Lackner^2^, Greta Haselrieder^3^, Thomas Müller^3^, Sabrina Sailer^4^, Johannes Zschocke^4^, Markus A. Keller^4^, A.S. Knisely^2,*^, Andreas R. Janecke^3,4,*^

^1^ Department of General Paediatrics and ^2^ Diagnostic and Research Institute of Pathology, Medical University of Graz, 8010 Graz, Austria; ^3^ Department of Paediatrics I and ^4^ Institute of Human Genetics, Medical University of Innsbruck, 6020 Innsbruck, Austria

**Supplemental methods**

**Liver-biopsy specimen processing**

Formalin-fixed and paraffin-processed tissue sections (4μm) from native-liver biopsy specimens (n=2; propositus and control) were routinely stained with haematoxylin / eosin (H&E), periodic acid - Schiff (PAS), Perls, chromotrope aniline blue, Gömöri reticulin, rhodanine, and Victoria blue techniques; diastase digestion before PAS staining also was employed. Formalin-fixed, paraffin-embedded liver, with same-slide control liver, was further immunostained (automated processing; Ventana Benchmark ULTRA, Roche Diagnostics, Indianapolis, IN) with an unconjugated polyclonal rabbit antibody against DECR1 (Atlas Antibodies, Stockholm, SE; HPA023238, 1:500) and separately with an unconjugated polyclonal rabbit antibody against DECR2 (Abcam, Cambridge, UK; ab153849, 1:50), for both using diaminobenzidine as chromogen and haematoxylin as counterstain.

Liver material (n=2; propositus, control) primarily fixed in 2.5% glutaraldehyde in cacodylate buffer at bedside on sampling was post-fixed (OsO_4_) and embedded in resin (AGAR 100 Resin R1031, Agar Scientific, Stansted, UK). After light microscopy of semi-thin sections (azur 2 / methylene blue), ultrathin sections stained with uranyl acetate / lead citrate were evaluated by transmission electron microscopy using a ZEISS EM900 TEM (Carl Zeiss, Oberkochen, Germany).

**Analysis of acylcarnitines**

The proband underwent blood and urine sampling after 12h, 18h, and 24h of a fast. Acylcarnitines were extracted from plasma (20µl) using 3:1 (v/v) acetonitrile : methanol. Internal acylcarnitine standards NSK-B-1 and NSK-B-G1-1 were purchased from Cambridge Isotope Laboratories (Tewksbury, MA). The internal-standard mixture was supplemented with malonyl-L-carnitine-(N-methyl-d3), hexanoyl-L-carnitine-(N-methyl-d3), and decanoyl-L-carnitine-(N-methyl-d3) (Sigma Aldrich, Vienna, Austria). Samples were processed by beat homogenization in a cryomill for 2.5min at 20 Hz, sonicated for 5min and centrifuged for 10min at 31,000 g. Supernatant was transferred to a glass vial and evaporated under a stream of nitrogen at 37°C. Acylcarnitines were quantified on an Elute UHPLC system (Bruker, Bremen, Germany) coupled to a timsTOf Pro ion-mobility mass spectrometer (Bruker). For hydrophilic-interaction liquid chromatography, 10µl of the extract was injected onto an Acquity UPLC® BEH HILIC 1.7 µm column (2.1 x 100 mm) (Waters, Milford, MA) with a column temperature of 30°C. The mobile phase consisted of (A) 100% water and (B) 90:10 (v/v) acetonitrile : water, each containing 0.1 % formic acid and 5mM ammonium formate. The flow rate was set to 0.4 ml/min. The liquid-chromatography gradient consisted of: isocratic at 98% B 0 min, ramp to 93% B at 0.5 min, ramp to 60% B at 7 min, ramp to 40% B at 9min, ramp to 98% at 10.5 min, and isocratic at 98% B for 11.5 - 12 min. The flow was increased to 0.5 ml/min at 9.5 - 10.5 min. Identification and peak integration of acylcarnitines was performed using TASQ® (Targeted Analysis Screening Quantitation; Bruker). Data were processed in R for metabolite quantification via isotope-labelled internal-standard responses. In addition to standard reporting of acylcarnitine species,[1] the following polyunsaturated long-chain species were determined: C20:1, C20:2, C20:3, C20:5, C22:0, C22:1, C22:2, C22:4, C22:5 und C22:6.

**Whole exome sequencing, sequence data analysis, and variant validation**

Genomic DNA was extracted from peripheral blood samples of participants using standard procedures. Human protein-coding genes (36.8 Mb in total) were captured from genomic DNA with the Twist Comprehensive Exome Panel and with the Mitochondrial Panel (Twist Bioscience, San Francisco, CA); reagents from the same kits were used to prepare DNA libraries, which were sequenced on a HiSeq platform (Illumina, San Diego, CA) with 150 bp read length in paired-end sequencing mode. The obtained sequencing reads were aligned to the human reference genome “Genome Reference Consortium Human Build 38 Organism: Homo sapiens (GRCh38)” (University of California Santa Clara, Santa Clara, CA) and variants were called with the Genome Analysis Toolkit (GATK) version 4.0 (<https://github.com/broadinstitute/gatk>). Sequencing reads were also aligned to the human reference genome “Genome Reference Consortium Human Build 37 Organism: Homo sapiens (GRCh37)” with SeqNext (Version 5.0; JSI, Kippenheim, Germany).

Called variants were filtered for autosomal recessive mode of inheritance (including both homozygous and compound heterozygous variants), for predicted effect on protein expression (missense, nonsense, intronic variants at exon-intron boundaries ranging from -15 to +15, in-frame indels, and frameshift), and for allele frequency of <0.005 in the gnomAD database (<https://gnomad.broadinstitute.org/>). Variants were evaluated *in silico* for pathogenicity by CADD (<http://cadd.gs.washington.edu/score>)[2]; missense variants were evaluated by PolyPhen-2 (<http://genetics.bwh.harvard.edu/pph2>)[3] and SIFT[4]; and splice site variants were evaluated using SpliceAI lookup (https://spliceailookup.broadinstitute.org/). Sanger sequencing of a genomic PCR fragment (forward primer 5`GAAATGCTTTTCTTTAAGCTATGGA, reverse primer 5`ATGACATTTTCCAGCTTTCTTAAA) permitted *DECR1* variant validation and segregation within the family. *DECR1* variant designation is based on two National Center for Biotechnology Information reference sequences for *DECR1* transcript *NM_001359.2*. Variants were classified according to the criteria proposed by the American College of Medical Genetics and Genomics [5].

**Exome data evaluation for known human fatty acid oxidation disorders and hepatopathies**

Pathogenic or likely pathogenic variants in genes associated with known human fatty acid oxidation disorders or hepatopathies (https://panelapp.genomicsengland.co.uk/panels/; panels: [Mitochondrial liver disease, including transient infantile liver failure,](https://panelapp.genomicsengland.co.uk/panels/532/) neonatal cholestasis, hyperammonaemia, and mitochondrial disorders) were not found in the propositus nor his siblings.

***DECR1* transcript analysis**

Using the Qiagen RNAeasy mini kit (Qiagen, Hilden*,* Germany), total RNA was extracted from EDTA-anticoagulated blood of the propositus, his parents, and a control, and cDNA was transcribed with SuperScript III (Invitrogen, Fisher Scientific, Vienna, Austria). To assess functional consequence of a variant in the *DECR1* intron 3 donor splice-site, denoted NG_008042.2(NM_001359.2):c.273_330del, a semi-quantitative reverse transcriptase (RT)-PCR reaction was performed with a forward primer binding to NM_001359.2 exon 2 (5`-ATGGGACAAAAATATTATATCAAAACA) and a reverse primer binding to exon 6 (5`-TTGCTCATGGCTTCCACAC). cDNA-derived PCR products were sequenced by conventional Sanger sequencing using Big Dye 149 terminator chemistry on an ABI PRISM 3100 Genetic Analyzer (Applied Biosystems, Fisher Scientific). Data were analyzed with JSI software (version 5.3).

**Polymorphisms associated with metabolic dysfunction-associated steatotic liver disease (MASLD)**

To identify potentially confounding factors for the hepatic phenotypes in patients II-1, II-2, and II-3, we assessed the 5 family members’ exomic genotypes at the sites of the most widely studied single nucleotide polymorphisms (SNPs, Table S3) that have been identified by genome-wide investigations as associated with the development of MASLD (Table S3).

**SUPPLEMENTAL RESULTS**

**Table S1. Hematologic, liver, and metabolic parameters, Patients II-3 (propositus) and II-2*, and their brother II-1. ****

|  |  | **Unit** | **At presentation** | | | **At last examination** | | |
| --- | --- | --- | --- | --- | --- | --- | --- | --- |
| **Patient** |  |  | II-3 | II-2 | II-1 (wt/wt) | II-3 | II-2 | II-1 (wt/wt) |
| **Age** |  |  | 6y | 11y | 14y | 10y | 13y | 16y |
| **BMI** |  |  | 17.1 | 26.3 | 31.2 | 22.0 | 26.6 | n.a. |
| **Hematologic** | **Hemoglobin** | g/dL | 12.1 | 13.1 | 12.7 | 13.1 | 12.8 | 15.1 |
| **parameters** | **Leukocytes** | G/L | 9.25 | 7.64 | 6.10 | 8.12 | 8.06 | 5.87 |
|  | **Thrombocytes** | G/L | 356 | 304 | 309 | 264 | 327 | 239 |
| **Liver** | **Total bilirubin** | mg/dL | 0.32 | 0.29 | 0.17 | 0.48 | 0.17 | 0.17 |
| **parameters** | **GGT** | U/L | 51 | **75** | 26 | **44** | **60** | 30 |
|  | **AP** | U/L | 193 | 385 | 321 | 249 | 306 | 111 |
|  | **ALT** | U/L | **203** | **61** | 28 | **101** | 29 | 35 |
|  | **AST** | U/L | **182** | **58** | 29 | 71 | 34 | 30 |
|  | **Albumin** | g/dL | 4.6 | 4.8 | 5.1 | 4.5 | 4.7 | 5.1 |
|  | **INR** |  | 1.06 | 0.98 | 0.89 | 1.10 | 0.98 | 0.95 |
|  | **aPTT** | sec | **27.2** | n.a. | 23.7 | 30.8 | 33.6 | 29.3 |
| **Metabolic** | **Cholesterol** | mg/dL | 100 | **253** | 177 | 119 | **273** | **231** |
| **parameters** | **Triglycerides** | mg/dL | 167 | **172** | 133 | 68 | **225** | 140 |
|  | **HDL** | mg/dL | **29** | 44 | 29 | **14** | 42 | 30 |
|  | **LDL** | mg/dL | 98 | 174 | 121 | 92 | 186 | 171 |
|  | **VLDL** | mg/dL | 16 | n.a. | n.a. | 12 | **37** | 29 |
|  | **HbA1c** | mmol/mol | 37 | **88** | n.a. | 40 | **67** | 42 |
|  | **Lysine** | µmol/L | 122.8 | n.a. | n.a. | 148 | 168 | 158 |

**Legend:** wt/wt, biallelic wild-type; n.a. – not available; * Burkitt lymphoma treatment protocol available at: *NHL-BFM Registry*, https://www.gpoh.de/studienportal/studien_und_register___onkologie/nhl_bfm_registry_2012/index_ger.html); ** Values exceeding age-normalized limits are **boldfaced** and underlined. Values for clinical-biochemistry parameters were ascertained by standard laboratory techniques.

**Table S2. Test results, 24h fast, Patient II-3.***

|  | |  |  |  |  |  |  |  |  |
| --- | --- | --- | --- | --- | --- | --- | --- | --- | --- |
| duration | glucose | pH | pCO2 | HCO3 | BE | lactate | β-hydroxybutyrate | glucose | ketones |
|  | *mg/dL* |  | *mmHg* | *mmol/L* | *mmol/L* | *mmol/L* | *mmol/L* | (urine) | (urine) |
| 12h | n.a. | n.a. | n.a. | n.a. | n.a. | 2.2 | n.a. | absent | absent |
| 18h | 90 | 7.392 | 39 | 23.7 | -1.1 | 1.5 | 0.15 | absent | absent |
| 24h | 85 | 7.393 | 39 | 23.8 | -1 | 1.4 | **0.51** | absent | **+++/++** |

**Legend:** n.a. – not available; * Values exceeding age-normalized limits are **boldfaced** and underlined.

**Table S3. Family members’ MASLD risk factors including genotypes at major published MASLD-associated polymorphic sites**

|  |  |  |  | **Risk factor** |  | **Family members’ risk factors**  ***** | | | | |
| --- | --- | --- | --- | --- | --- | --- | --- | --- | --- | --- |
|  |  |  |  |  |  | **II-3** | **II-2** | **II-1** | **I-1** | **I-2** |
|  |  |  |  | **obesity** |  | Normal weight | **Class**  **I**  **obesity** | **Class**  **II**  **obesity** | Normal weight | Normal weight |
|  |  |  |  | **Diabetes mellitus type 2** |  | No | **Yes** | No | No | **Yes** |
|  |  |  |  | **Chemotherapy, Burkitt lymphoma, stage 2^#^** |  | No | **Yes** | No | No | No |
|  |  |  |  |  |  |  |  |  |  |  |

| **Gene** | **SNP** ID | **SNP localization**  **GRCh38**  **(GRCh37)** | **SNP** consequence | **Correlation of alternate allele with MASLD** | **Refs.** |  |  |  |  |  |
| --- | --- | --- | --- | --- | --- | --- | --- | --- | --- | --- |
| DECR1 | rs371896281 | 8-90018969-A-T  (8-91031197-A-T ) | ENST00000220764.7  c.330+3A>T | Promote | **This**  **Study ^##^** | **TT** | **TT** | **AA** | AT | AT |
| PNPLA3 | rs738409 | 22-43928847-C-G  (22-44324727-C-G) | ENST00000216180.8p.Ile148Met | Promote | [6] | GG | GG | GG | GG | CG |
| PNPLA3 | rs3747207 | 22-43928975-G-A (22-44324855-G-A) | ENST00000216180.8 c.486+86G>A | Promote | [7] | AA | AA | AA | AA | GA |
| PNPLA3 | rs2896019 | 22-43937814-T-G(22-44333694-T-G) | ENST00000216180.8c.979+542T>G | Promote | [8] | GG | GG | GG | GG | TG |
| TM6SF2 | rs58542926 | 19-19268740-C-T (19-19379549-C-T) | ENST00000389363.5  p.Glu167Lys | Promote | [9] | CC | CC | CC | CC | CC |
| SUGP1 | rs10401969 | 19-19296909-T-C(19-19407718-T-C) | ENST00000247001.10  c.1243+80A>G | Promote | [10] | TT | TT | TT | TT | TT |
| HSD17B13 | rs72613567 | 4-87310240-T-TA (4-88231392-T-TA) | [ENST00000328546.5](https://gnomad.broadinstitute.org/transcript/ENST00000328546?dataset=gnomad_r4)c.812+2dup | Protect | [11] | T/TA | T/TA | T/TA | T/T | T/TA |
| HSD17B13 | rs13118664 | 4-87318457-A-T (4-88239609-A-T) | [ENST00000328546.5](https://gnomad.broadinstitute.org/transcript/ENST00000328546?dataset=gnomad_r4)c.211-21T>A | Protect | [12] | AT | AT | AT | AA | AT |
| HSD17B13 | rs9992651 | 4-87311358-G-A (4-88232510-G-A) | [ENST00000328546.5](https://gnomad.broadinstitute.org/transcript/ENST00000328546?dataset=gnomad_r4)c.696-999C>T | Protect | [12] | n.a. | n.a. | n.a. | n.a. | n.a. |
| MTARC1 (MARC1) | rs2642438 | 1-220796686-A-G(1-220970028-A-G) | [ENST00000366910.10](https://gnomad.broadinstitute.org/transcript/ENST00000366910?dataset=gnomad_r4)p.Thr165Ala | Protect | [13] | **AG** | **AA** | **GG** | AG | AG |
| GCKR | rs780094 | 2-27518370-T-C  (2-27741237-T-C) | [ENST00000264717.7](https://gnomad.broadinstitute.org/transcript/ENST00000264717?dataset=gnomad_r4)  c.1423-418T>C | Promote | [14] | CC | CC | CC | CC | CC |
| GCKR | rs1260326 | 2-27508073-T-C  (2-27730940-T-C) | [ENST00000264717.7](https://gnomad.broadinstitute.org/transcript/ENST00000264717?dataset=gnomad_r4)  p.Leu446Pro | Promote | [15] | CC | CC | CC | CC | CC |
| TMC4 / MBOAT7 ** | rs626283 | 19-54173307-C-G (19-54677001G>C) | TMC4 2kb Upstream Variant / MBOAT7 500b downstream Variant | Promote | [16, 17] | GG | GC | GC | GC | GG |
| TMC4 / MBOAT7 ** | rs641738 | 19-54173068-T-C  (19-54676763C>T) | ENST00000619895.5  TMC4 p.Glu17Gly / MBOAT7 downstream variant | Promote | [18] | CC | CT | CT | CT | CC |
| HFE | rs1800562 | 6-26092913-G-A  (6-26093141-G-A) | ENST00000357618.10  p.Cys282Tyr | Promote*** | [19] | GG | GG | GG | GG | GG |
| SLC17A3-SLC17A2 | rs9348697 | 6-25890606-C-T  (6-25890834-C-T) | Intergenic variant | Promote *** | [19] | n.a. | n.a. | n.a. | n.a. | n.a. |

**Legend:** n.a. – not available; ^#^ Chemotherapy of II-2 consisted of: initiation block with: dexamethasone, cyclophosphamide, MTX, cytarabine und prednisolone; 4 therapy blocks of: dexamethasone, cyclophosphamide, MTX, cytarabine und prednisolone, vincristine, doxorubicine; plus one dosage of rituximab; GCKR, glucokinase regulator; HSD17B13, 17β-hydroxysteroid dehydrogenase 13; MTARC1, mitochondrial amidoxime reducing component 1; MARC1, mitochondrial amidoxime-reducing component 1; MBOAT7, membrane-bound O-acyltransferase 7; MASLD, Metabolic dysfunction–associated steatotic liver disease; PNPLA3, patatin-like phospholipase domain containing 3; SNPs, single nucleotide polymorphisms; SUGP1, SURP and G-patch domain containing 1; TM6SF2, transmembrane 6 superfamily member 2; TMC4, transmembrane channel like 4.

* Genotypes differing among children are **boldfaced** and underlined. **^##^**  Candidate variant implicated by this study.

** SNPs were associated with a modest risk of developing NAFLD (OR _~_1.37), NASH, and fibrosis in one cohort of patients of European descent, while not being significantly thus associated in African American and Hispanic populations.^[16]^ The association of rs641738 and NAFLD could not be replicated in other populations around the world, including Europeans from different cohorts.^[17]^

*** associated with non-alcoholic fatty liver disease and hemochromatosis

**Supplemental references**

1. Miller MJ, Cusmano-Ozog K, Oglesbee D, Young S, Committee ALQA. Laboratory analysis of acylcarnitines, 2020 update: a technical standard of the American College of Medical Genetics and Genomics (ACMG). Genet Med. 2021;23(2):249-58.

2. Rentzsch P, Witten D, Cooper GM, Shendure J, Kircher M. CADD: predicting the deleteriousness of variants throughout the human genome. Nucleic Acids Res. 2019;47(D1):D886-D94.

3. Adzhubei IA, Schmidt S, Peshkin L, Ramensky VE, Gerasimova A, Bork P, et al. A method and server for predicting damaging missense mutations. Nat Methods. 2010;7(4):248-9.

4. Ng PC, Henikoff S. SIFT: Predicting amino acid changes that affect protein function. Nucleic Acids Res. 2003;31(13):3812-4.

5. Richards S, Aziz N, Bale S, Bick D, Das S, Gastier-Foster J, et al. Standards and guidelines for the interpretation of sequence variants: a joint consensus recommendation of the American College of Medical Genetics and Genomics and the Association for Molecular Pathology. Genet Med. 2015;17(5):405-24.

6. Romeo S, Kozlitina J, Xing C, Pertsemlidis A, Cox D, Pennacchio LA, et al. Genetic variation in PNPLA3 confers susceptibility to nonalcoholic fatty liver disease. Nat Genet. 2008;40(12):1461-5.

7. Namjou B, Lingren T, Huang Y, Parameswaran S, Cobb BL, Stanaway IB, et al. GWAS and enrichment analyses of non-alcoholic fatty liver disease identify new trait-associated genes and pathways across eMERGE Network. BMC Med. 2019;17(1):135.

8. Islek EE, Sazci A, Ozel MD, Aygun C. Genetic variants in the PNPLA3 gene are associated with nonalcoholic steatohepatitis. Genet Test Mol Biomarkers. 2014;18(7):489-96.

9. Kozlitina J, Smagris E, Stender S, Nordestgaard BG, Zhou HH, Tybjaerg-Hansen A, et al. Exome-wide association study identifies a TM6SF2 variant that confers susceptibility to nonalcoholic fatty liver disease. Nat Genet. 2014;46(4):352-6.

10. Wang J, Conti DV, Bogumil D, Sheng X, Noureddin M, Wilkens LR, et al. Association of genetic risk score with NAFLD in an ethnically diverse cohort. Hepatol Commun. 2021;5(10):1689-703.

11. Pirola CJ, Garaycoechea M, Flichman D, Arrese M, San Martino J, Gazzi C, et al. Splice variant rs72613567 prevents worst histologic outcomes in patients with nonalcoholic fatty liver disease. J Lipid Res. 2019;60(1):176-85.

12. Anstee QM, Darlay R, Cockell S, Meroni M, Govaere O, Tiniakos D, et al. Genome-wide association study of non-alcoholic fatty liver and steatohepatitis in a histologically characterised cohort(☆). J Hepatol. 2020;73(3):505-15.

13. Emdin CA, Haas ME, Khera AV, Aragam K, Chaffin M, Klarin D, et al. A missense variant in Mitochondrial Amidoxime Reducing Component 1 gene and protection against liver disease. PLoS Genet. 2020;16(4):e1008629.

14. Speliotes EK, Yerges-Armstrong LM, Wu J, Hernaez R, Kim LJ, Palmer CD, et al. Genome-wide association analysis identifies variants associated with nonalcoholic fatty liver disease that have distinct effects on metabolic traits. PLoS Genet. 2011;7(3):e1001324.

15. Chahal D, Sharma D, Keshavarzi S, Arisar FAQ, Patel K, Xu W, et al. Distinctive clinical and genetic features of lean vs overweight fatty liver disease using the UK Biobank. Hepatol Int. 2022;16(2):325-36.

16. Mancina RM, Dongiovanni P, Petta S, Pingitore P, Meroni M, Rametta R, et al. The MBOAT7-TMC4 Variant rs641738 increases risk of nonalcoholic fatty liver disease in individuals of European descent. Gastroenterology. 2016;150(5):1219-30 e6.

17. Sookoian S, Flichman D, Garaycoechea ME, Gazzi C, Martino JS, Castano GO, et al. Lack of evidence supporting a role of TMC4-rs641738 missense variant-MBOAT7- intergenic downstream variant-in the susceptibility to nonalcoholic fatty liver disease. Sci Rep. 2018;8(1):5097.

18. Teo K, Abeysekera KWM, Adams L, Aigner E, Anstee QM, Banales JM, et al. rs641738C>T near MBOAT7 is associated with liver fat, ALT and fibrosis in NAFLD: A meta-analysis. J Hepatol. 2021;74(1):20-30.

19. Sun Z, Pan X, Tian A, Surakka I, Wang T, Jiao X, et al. Genetic variants in HFE are associated with non-alcoholic fatty liver disease in lean individuals. JHEP Rep. 2023;5(7):100744.
